# Supplementary material for: Emerging experience-dependent dynamics in primary somatosensory cortex reflect behavioral adaptation
Source: Nat Commun. 2022 Jan 27;13:534. doi: 10.1038/s41467-022-28193-z (PMC8795122; doi:10.1038/s41467-022-28193-z)
Supplement: Supplementary file 1 — Supplementary Information [file 41467_2022_28193_MOESM1_ESM.pdf]

Waiblinger et al., Emerging experience-dependent dynamics in primary somatosensory cortex reflect behavioral adaptation

Supplemental Figures

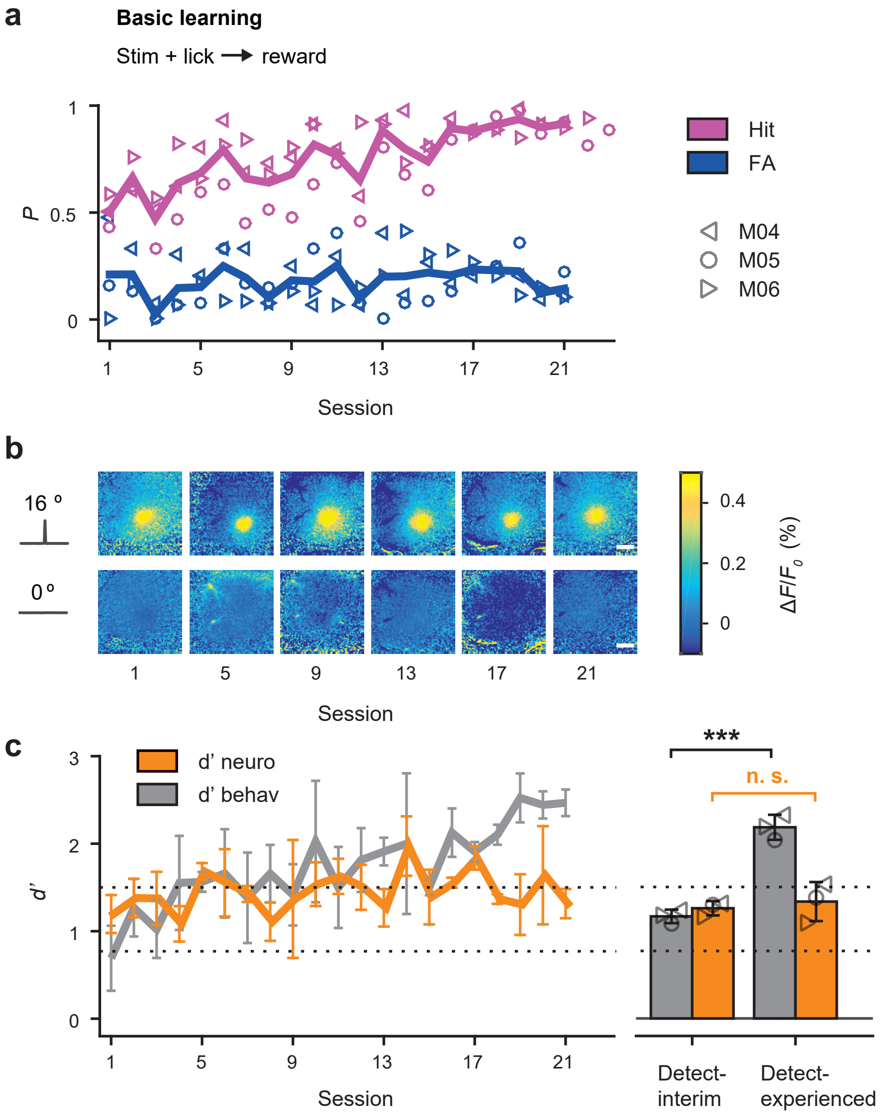

Supplemental Figure 1. Basic Learning with a strong stimulus. a, Learning curve for 3 mice trained on the basic Go/No-Go detection task with a strong stimulus (16 degree). **b,** Fluorescent activity in S1 from an example mouse during learning. Shown are frames at the peak response to a 16 degree stimulus, catch trials are shown below. Scale bar: 1 mm. **c,** Dprime metrics for both behavioral and neuronal data during learning of the task. Note, mice achieved higher hit rates at the beginning of training and reached successful task acquisition in half the time if compared to mice detecting a weak stimulus (Fig. 2). The dotted lines separate performance into "detect-interim" (*d'*=0.8-1.5) and "detect-experienced" (*d'*>1.5). The right panel shows the same data separated for individuals (symbols) before and after learning. Bars represent means across mice, error bars represent SD (*n*=3). *** *P*<0.001, n. s. not significant, *P*=0.21, two-sided Wilcoxon rank-sum test. Otherwise, figure conventions are the same as in Figure 2.

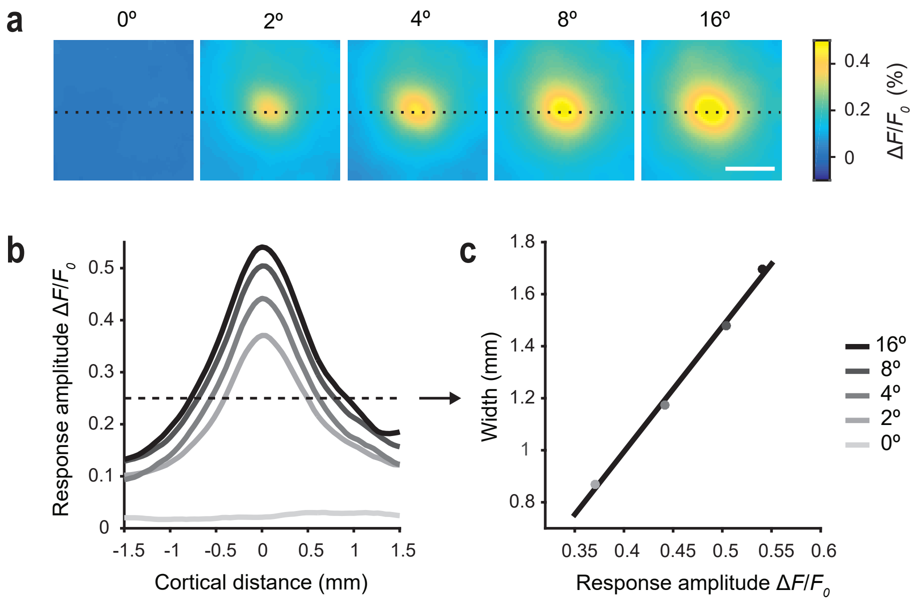

Supplemental Figure 2. The relationship of magnitude and activated area in GEVI imaging. a, Fluorescence activity in response to different stimuli. Each frame is normalized to the frame at stimulus delivery ($\Delta F/F_0 = F\text{-}F_0/F_0$). Shown are images at the response peak. The dotted line represents a slice through the images aligned with the maximum fluorescence. Scale bar: 1 mm. **b,** Magnitude (response amplitude $\Delta F/F_0$) versus cortical area extracted from the slice in a. Activity patterns are shown in different shades of grey for different stimulus amplitudes. **c,** Relationship between fluorescence magnitude and width of activated area, both scale with stimulus strength in a highly correlated fashion. The width is derived from a threshold (dashed line in b). Data points correspond to different stimulus amplitudes fitted with a linear regression. Shown are means across mice (n=4) and sessions (n=40).

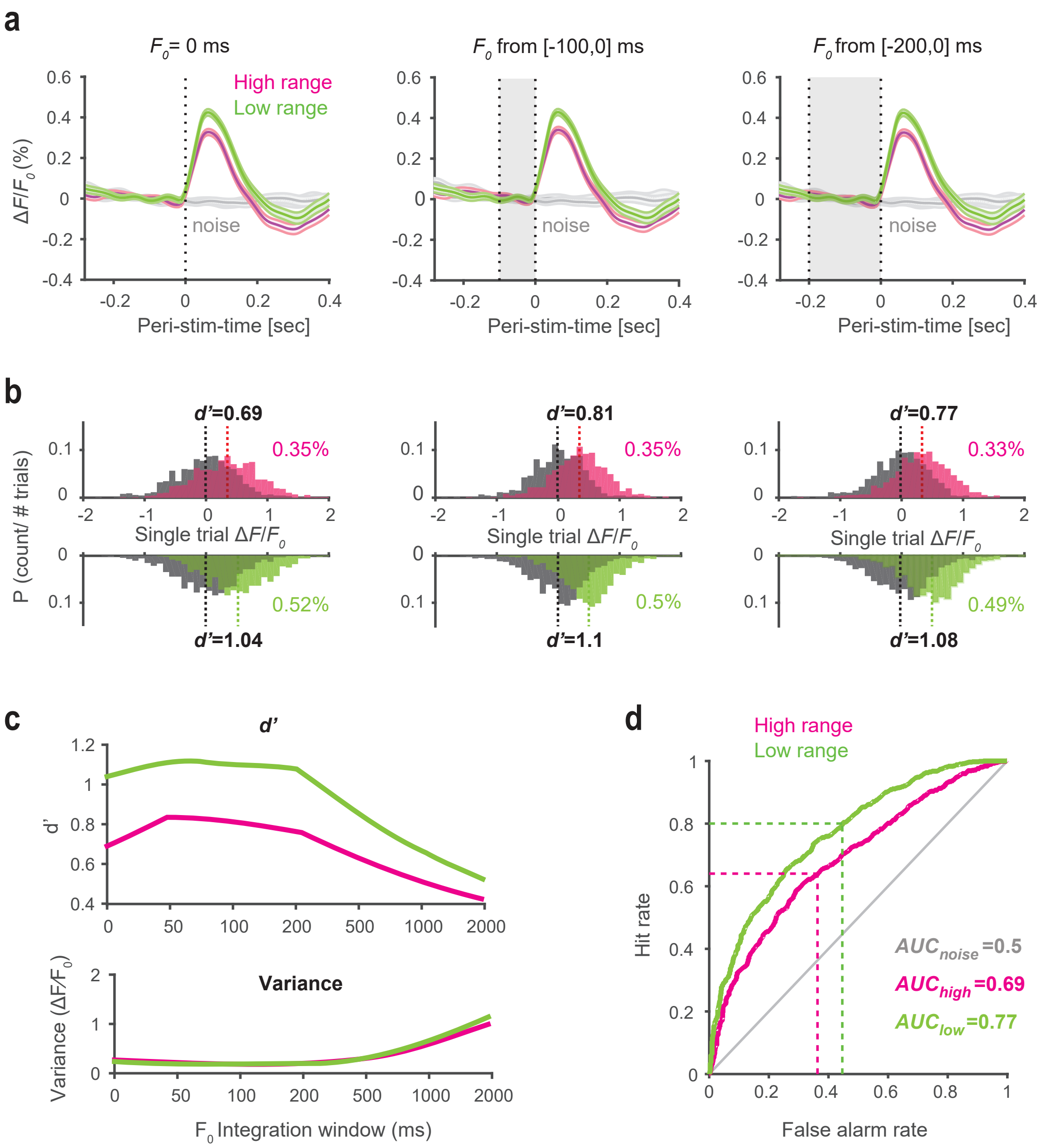

Supplemental Figure 3. Analysis to test stability of adaptive S1 response. a, Different normalization methods. F_0 was varied by using the mean fluorescence across different time windows before stimulus onset. The ΔF/F_0 measurement was then calculated by F-F_0/F_0. Mean fluorescence traces (*n*=4 mice, 831-870 trials) in response to the high (magenta) and the low range (green) stimulus condition. Error bands represent SEM. The grey box depicts the window for calculating F_0. From left to right: F_0=0 ms (frame at stimulus delivery), F_0 from [-100,0] ms, and F_0 from [-200,0] ms. **b,** Single-trial signal-peak and noise distributions from the data in a. Magenta and green numbers are mean fluorescent values in % ΔF/F_0 for the high and low range condition respectively. Black numbers represent dprime metrics *d'*. **c,** *d'* and variance for different F_0 calculations. **d,** Receiver operating characteristic (*ROC*) curves created by shifting the criterion across the ΔF/F_0 signal and noise distributions of the high and low range condition. The downstream criterion *c* can be inferred by comparing the hit rate in *ROC* space with the average behavioral hit rate (dashed lines).

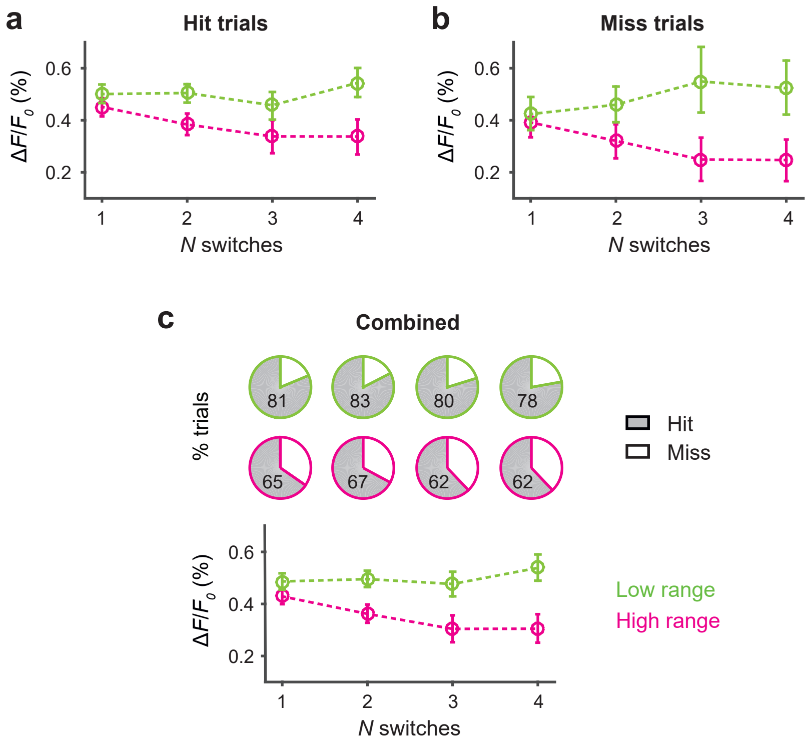

Supplemental. Figure 4. Choice-related long-term S1 dynamics. a, GEVI peak responses (maximum % $\Delta F/F_0$) for correct choices (hit trials) to 8 degree stimulation. Data is shown over multiple switches for the high- and low range condition. **b,** GEVI responses for incorrect choices (miss trials). **c,** Top: Percentage of hit and miss trials for the high- and low range condition. Bottom: Combined GEVI data (hits+misses) as a function of switches. All data shown are bootstrapped estimates of means and 95% confidence limits (based on single trials from n=6 mice, n_{boot}=1000 repetitions).
