# Supplementary material for: Emerging experience-dependent dynamics in primary somatosensory cortex reflect behavioral adaptation
Source: Nat Commun. 2022 Jan 27;13:534. doi: 10.1038/s41467-022-28193-z (PMC8795122; doi:10.1038/s41467-022-28193-z)
Supplement: Supplementary file 2 — Reporting Summary [file 41467_2022_28193_MOESM2_ESM.pdf]

nature portfolio

Corresponding author(s): Garrett B Stanley

Last updated by author(s): 12/06/2021

Reporting Summary

Nature Portfolio wishes to improve the reproducibility of the work that we publish. This form provides structure for consistency and transparency in reporting. For further information on Nature Portfolio policies, see our Editorial Policies and the Editorial Policy Checklist.

Statistics

For all statistical analyses, confirm that the following items are present in the figure legend, table legend, main text, or Methods section.

n/a	Confirmed	
☐	☒	The exact sample size (*n*) for each experimental group/condition, given as a discrete number and unit of measurement
☐	☒	A statement on whether measurements were taken from distinct samples or whether the same sample was measured repeatedly
☐	☒	The statistical test(s) used AND whether they are one- or two-sided<br>*Only common tests should be described solely by name; describe more complex techniques in the Methods section.*
☐	☒	A description of all covariates tested
☒	☐	A description of any assumptions or corrections, such as tests of normality and adjustment for multiple comparisons
☐	☒	A full description of the statistical parameters including central tendency (e.g. means) or other basic estimates (e.g. regression coefficient) AND variation (e.g. standard deviation) or associated estimates of uncertainty (e.g. confidence intervals)
☐	☒	For null hypothesis testing, the test statistic (e.g. *F*, *t*, *r*) with confidence intervals, effect sizes, degrees of freedom and *P* value noted<br>*Give P values as exact values whenever suitable.*
☒	☐	For Bayesian analysis, information on the choice of priors and Markov chain Monte Carlo settings
☒	☐	For hierarchical and complex designs, identification of the appropriate level for tests and full reporting of outcomes
☐	☒	Estimates of effect sizes (e.g. Cohen's *d*, Pearson's *r*), indicating how they were calculated

Our web collection on statistics for biologists contains articles on many of the points above.

Software and code

Policy information about availability of computer code

Data collection	Behavior set up was programmed as a custom software written in Matlab and Simulink (Ver. 2015b; The MathWorks, Natick, Massachusetts, USA), which is available upon request. Voltage fluorescent data was collected using MiCAM05-N256 (Scimedia, Ltd) software.
Data analysis	Open-source software was used for Image data processing (MiCAM05-N256, Scimedia, Ltd). Psychometric data analysis and curve fits were performed using open-source software psignifit toolbox version 2.5.6 for MATLAB version 5 and up (Wichmann & Hill, 2001 a,b). All other analyses were performed with custom MATLAB programs, which is available in “Dryad” with the identifier https://doi.org/10.5061/dryad.h18931zmm (not live until dataset is published). The dataset can be accessed before final publication using the following link: https://datadryad.org/stash/share/peEgYEUyOWk_BdK_HU2R9nOld7xwSkIJv0JPtEE8gls

For manuscripts utilizing custom algorithms or software that are central to the research but not yet described in published literature, software must be made available to editors and reviewers. We strongly encourage code deposition in a community repository (e.g. GitHub). See the Nature Portfolio guidelines for submitting code & software for further information.

Data

Policy information about availability of data

All manuscripts must include a data availability statement. This statement should provide the following information, where applicable:

- Accession codes, unique identifiers, or web links for publicly available datasets
- A description of any restrictions on data availability
- For clinical datasets or third party data, please ensure that the statement adheres to our policy

The data that support the findings of this study are available in “Dryad” with the identifier https://doi.org/10.5061/dryad.h18931zmm (not live until dataset is

published). The dataset can be accessed before final publication using the following link: https://datadryad.org/stash/share/peEgYEUyOWk_BdK_HU2R9nOld7xwSkIJv0JPtEE8gls

Field-specific reporting

Please select the one below that is the best fit for your research. If you are not sure, read the appropriate sections before making your selection.

☒ Life sciences ☐ Behavioural & social sciences ☐ Ecological, evolutionary & environmental sciences

For a reference copy of the document with all sections, see nature.com/documents/nr-reporting-summary-flat.pdf

Life sciences study design

All studies must disclose on these points even when the disclosure is negative.

Sample size	Sample sizes were chosen in accordance with the standard number of animals in comparable studies in the field (e.g. Banerjee et al. 2020 Nature). A total of n = 13 mice were used in tis study with n = 2-6 mice per group in each imaging, behavior or lesion experiment. The exact number of animals for specific groups are given in the main text and also in figure legends.
Data exclusions	Mice that did not express the genetically encoded voltage fluorescent protein (GEVI) or developed bleeding or opaque skull during the behavioral training period and longitudinal imaging were excluded from the analyses. Trials with premature licking in a 2 s period before the stimulus delivery were excluded from the main data analysis. The exact criteria for exclusion of these trials is given in the Methods section.
Replication	All experiments were reproduced multiple times in multiple animals. The results of the original dataset (n = 7 mice) were replicated by adding more animals (n = 13 total) and two more experiments (1. repeated conditions, 2. lesion and sham lesions) upon reviewer requests. All attempts of replication were successful.
Randomization	For the main part of the study (Fig. 3, 4 and 5) animals served as their own control, so randomization would not be relevant. For the basic learning experiments (Fig. 2) and lesion experiments (Fig. 6), animals were allocated randomly into experimental groups. Within behavior and imaging sessions, stimulus presentation was randomized and inter-trial intervals were drawn from at random from a flat probability distribution.
Blinding	All animal subjects were handled and trained exactly the same way despite differences in experimental conditions. Experiments were run automatically by a computer with predefined parameters, which removes any subjective component of the data collection. Furthermore, the data was analyzed with equal parameters across subjects and conditions. Otherwise, no blinding was performed as this is not strictly relevant to this study.

Reporting for specific materials, systems and methods

We require information from authors about some types of materials, experimental systems and methods used in many studies. Here, indicate whether each material, system or method listed is relevant to your study. If you are not sure if a list item applies to your research, read the appropriate section before selecting a response.

Materials & experimental systems

n/a	Involved in the study	
☒	☐	Antibodies
☒	☐	Eukaryotic cell lines
☒	☐	Palaeontology and archaeology
☐	☒	Animals and other organisms
☒	☐	Human research participants
☒	☐	Clinical data
☒	☐	Dual use research of concern

Methods

n/a	Involved in the study	
☒	☐	ChIP-seq
☒	☐	Flow cytometry
☒	☐	MRI-based neuroimaging

Animals and other organisms

Policy information about studies involving animals; ARRIVE guidelines recommended for reporting animal research

Laboratory animals	Subjects were thirteen male mice (C57BL/6, Jackson Laboratories), aged 4-6 weeks at time of implantation. Detailed information on housing conditions is given in the main text of the manuscript.
Wild animals	The study did not involve wild animals.
Field-collected samples	The study did not involve field-collected samples.

Ethics oversight	All experimental and surgical procedures were approved by the Georgia Institute of Technology Institutional Animal Care and Use Committee and were in agreement with guidelines established by the NIH.

Note that full information on the approval of the study protocol must also be provided in the manuscript.
